# Observed Dietary Intake in Adults with Intellectual Disability Living in Group Homes

**DOI:** 10.3390/nu12010037

**Published:** 2019-12-22

**Authors:** Nur Hana Hamzaid, Helen T. O’Connor, Victoria M. Flood

**Affiliations:** 1 Faculty of Health Sciences, Universiti Kebangsaan Malaysia, Program of Dietetics, Kuala Lumpur 50300, Malaysia; hanahamzaid@ukm.edu.my; 2 Faculty of Health Sciences, The University of Sydney, Discipline of Exercise and Sport Science, Lidcombe 2141, NSW, Australia; helen.oconnor@sydney.edu.au; 3 Westmead Hospital, Western Sydney Local Health District, Westmead 2145, NSW, Australia; 4 Charles Perkins Centre, The University of Sydney, Camperdown 2006, NSW, Australia

**Keywords:** dietary intake, intellectual disability, group homes, macronutrients, micronutrients

## Abstract

Background: There is limited information on the dietary intakes of people with intellectual disability (ID) living in group homes. Objective: To describe and evaluate dietary intake in people with ID. Method: Dietary intake was assessed in a convenience sample of people with ID living in group homes. Dietary assessment used three-day weighed food records and digital food photography. Intakes were compared to the Nutrient Reference Values (NRVs) and dietary recommendations. Results: A sample of 33 adults, (men (M): *n* = 14; women (W): *n* = 19), mean age 51 ± 14 years, was recruited from seven group homes. Mean daily energy intake was low (M: 7.4 MJ; W: 7.0 MJ; *p* = 0.46), similar to levels recommended for bed rest. Many participants had intakes below the estimated average requirements (EARs) for the nutrients, magnesium (M: 86%; W: 63%), calcium (M: 43%; W: 78%), iodine (M: 43%; W: 47%) and zinc (M: 43%). Less than half of the recommended daily servings were consumed for vegetables (men and women) and dairy foods (women). Conclusion: Nutrient intake and diet quality of the participants in the group homes studied was poor. Education and policy to support healthier diets is required to improve dietary intake of people with intellectual disability, living in group homes.

## 1. Introduction

Internationally, policies informing residential support for persons with intellectual disability (ID) encourage community rather than institutional living [1,2,3]. These policies aim to facilitate the creation of an environment for people with ID to support inclusion and self-determination [4,5,6,7]. Currently, the preferred option for community accommodation in Australia and internationally is small-scale group homes [8]. This type of care has been shown to result in consistent improvement in adaptive behaviour, competence and personal growth [6,9,10]. It also fosters community participation, engagement in meaningful activities and enhanced contact with staff, family, friends and other social networks. The result is higher client, parent and staff satisfaction with greater self-determination and quality of life for people with ID [5,6,7,11]. Of all the individuals with ID in Australia, approximately 77.2% live in small group homes [12].

In Australia, approximately 3% of the population are diagnosed with ID and most are aged under 65 years, as life expectancy in this population is lower than the general community [13,14]. Although decreased life expectancy in people with ID is partly due to congenital medical problems associated with ID syndromes, lifestyle-related conditions including obesity, type 2 diabetes and cardiovascular disease are also more prevalent in people with ID [15,16,17]. Adoption of detrimental lifestyle behaviours such as consumption of high-saturated fat, high-salt, low-fibre foods, poor calcium intake, and inadequate intake of fruit and vegetables combined with low sunlight exposure and low levels of physical activity is known to increase the risk of lifestyle-related chronic conditions [18,19,20].

Commonly used medications may also be associated with negative side effects that translate to an increased risk for lifestyle disease (e.g., weight gain secondary to the use of anti-depressants) in people with ID [21]. Specific conditions such as Prader Willi syndrome alter appetite and increase the risk for weight gain and obesity [22,23,24]. Conversely, a reduced capacity to chew and swallow may make eating more difficult and require the texture of foods to be modified. While a nutritious, texture modified diet can be prepared, this may be less convenient and more labour intensive for carers. Some people with ID may also require assistance with feeding (cutting up food or need to be fed) and lack of adequate assistance for this can negatively impact dietary intake [25].

Additionally, people with ID may have a diminished ability to comprehend and assess information about nutrition, leading to poor dietary choices [26]. They may also have less control over food purchases and preparation and if these are not aligned with healthy recommendations, the risk of nutrition-related disease is increased [27,28]. Conversely, it has been proposed that with a less restrictive environment in community, people with ID are more exposed to a wider range of food habits and some of these may potentially exacerbate the development of lifestyle diseases [29,30]. Internationally, the transition from institution to community living has been relatively rapid and research has not yet addressed the full impact of this change, so there is limited research on the dietary intake of people with ID living in the community [4,26,31]. Carers in the community living residents were also found to have limited nutrition knowledge that may compromise their ability to plan and adapt healthy meal for people with ID in their residence [32].

Dietary intake has significant potential to optimise health and modify the risk of lifestyle disease [33,34,35]. The New South Wales Agency for Clinical Innovations documented the Nutrition Standards for Consumers of Inpatient Mental Health Services document [36], which states the importance of providing adequate nutritional intake to consumers, and both the World Health Organisation and the American Dietetic Association have supported the need to improve the nutritional status of people with ID [37,38]. Studies on dietary intake and the food provision system of people with ID living in group homes in Australia have been limited. Hence, the current study aimed to evaluate the dietary intake of people with ID residing in a sample of group homes that provided all meals in Sydney, Australia.

## 2. Materials and Methods

### 2.1. Participants, Setting and Design

The sample of organisations was recruited from not-for-profit organisations that provided group home facilities where the research team had established professional links. Invitations were sent to three not-for-profit organisations that had group homes operating in a geographical perimeter feasible for the team to conduct the research (within a 40 km radius). To be included, the group homes needed to provide all meals (including between meal snacks and beverages) to participants.

Eligible participants were residents with mild to profound ID [39] living in the group homes of the organisations recruited. The classification of ID was made by physicians and health practitioners seen by each participant prior to their move to the group homes.

To be included in the study, adult residents, aged ≥18 years, needed to be able swallow solid food (including texture modified: chopped, minced or pureed foods), and not be completely dependent on supplemental nutrition (e.g., naso-gastric feeding). Residents requiring partial (≤500 mL/day) supplementation via a commercial formula were eligible for inclusion. This study had a cross-sectional design with recruitment and data collection of anthropometric and dietary intake data occurring once for each participant, in 2012 to 2013. The study was approved by The University of Sydney Human Ethics Committee. Informed consent was provided by either the participant, parents or legal guardians.

### 2.2. Anthropometric Measurements

Baseline anthropometric measurements were taken by a research dietitian (NHH). Body weight (accurate to 100 g) was measured in light clothing (no shoes) using a digital platform scale (Tanita Body Composition Monitor Model BC-541). Stature (accurate to 0.1 cm) was measured without shoes using a wall-mounted stadiometer (Surgical and Medical Products^TM^). When measurement was not possible (due to refusal or physical disabilities), the most recent anthropometric data was obtained from participant medical records. Participant BMI was classified according to Australian guidelines [40].

### 2.3. Measurement and Assessment of Dietary Intake

Dietary intake was measured over three days (two weeks and one week-end day) via a proxy-reporter method [4], where a weighed food record was collected by a research dietitian (NHH), supplemented with photos. The days chosen for observation were based on convenience of the home and researchers, but care was taken to identify usual or typical days for the recording period. Each food was weighed (household scale accurate to 1 g) and all waste was weighed to enable calculation of the edible portion. This method was chosen to enhance accuracy and to accommodate any possible limitations with the participants recording their own food intake and also to reduce recording burden for the carers [4,18,41,42].

Group home meals were prepared and plated in a domestic kitchen setting by paid carers. Participants were assisted with food consumption (e.g., food cut up or fed) as required by the carers who were present at all home meals. If participants were engaged in day programs outside the home, morning tea and lunch was prepared and pre-packed by the carer (s) on duty. In this instance, the carers were trained and instructed to record the food consumed using a weighed record or if this was not possible, a written record describing the foods consumed in household measures.

The carers were also instructed to take a digital photograph on 2 × 2 cm grid-style placemat labelled with the participant’s name to support the weighed or household measure record or to be used alone if neither of the above methods were practical. All foods were digitally photographed before and after the meal on a 2 × 2 cm grid placemat which were used as a ‘scale’ to calculate the meal size followed by a verbal reporting to the researcher in the following visit. The gridded placemat has a space for writing identification note to identify the mealtime and to whom the meal belonged. Left-over food not consumed by the participants at work or day programs was weighed by the research dietitian (NNH) when they returned to the group home in the afternoon. On-duty carers were also given the responsibility to record food during unscheduled mealtime, for example during unplanned snack time at home. Similar methodology using digital photography has been previously shown to enhance the quality of diet records in a similar setting [43,44,45].

Computerised diet analysis of the menus offered, and all the weighed food of individual intakes was conducted using the dietary analysis software package FoodWorks (Version 6.0.25175; Xyris Software, Brisbane, Australia), using the nutrient database of Australian foods [46]. When weight was not available from the weighed food record, the weight was estimated based on ratio of the photos to the standard size, as reported in the nutrient database used in the FoodWorks program. The photos were taken on a grid which supported the estimate of the food items. There were also times where the weights were supported from images of the nutritional information panel of the food items consumed. Energy and nutrients provided by commercial supplemental formulas were included (for individual intake). Folate and iodine values were adjusted to reflect folate fortified food products, in particular for bread, using a more updated database [47]. Salt added to the recipes prepared by carers was included in the analysis. Discretionary salt was deemed negligible, as salt was not provided at the dining table at any of the group homes.

### 2.4. Assessment of the Adequacy of Dietary Intake

Dietary intakes were compared against Nutrient Reference Values (NRVs) [48] and the Australian Guide to Healthy Eating (AGHE) for Australian adults [40]. Specifically, this included comparison to the Recommended Dietary Intake (RDI) (i.e., considered a level adequate for 97%–98% of healthy individuals within specific age and gender groups), the Estimated Average Requirement (EAR) (i.e., considered a level adequate for 50% of healthy individuals within specific age and gender groups) and upper limit (UL) (i.e., the highest average daily nutrient limit likely to pose no adverse health effects to most people). When an RDI was not available for a nutrient, an Adequate Intake (AI) was used [48]. Macronutrients were compared to the Australian Acceptable Macronutrient Distribution Ranges (AMDRs) recommended for the population. The intake of dietary fibre and sodium was compared to the Suggested Dietary Targets (SDTs) (i.e., a daily average intake from food and beverages for nutrients that that may help in prevention of chronic disease) [48].

The use of established equations (e.g., Harris Benedict 1919, Schofield 1985) to estimate energy requirements from resting metabolic rate of the participants (using measured weight, height, age, etc.) was considered inappropriate, as these equations validated on healthy individuals have been shown to overestimate energy requirements in individuals with ID [18]. This is because individuals with ID have lower lean mass than populations without ID and lean mass is a key predictor of resting energy expenditure [18]. Evidence also suggests that people with ID are more sedentary than those without ID [4,18]. Although the literature is limited, existing evidence indicates that people with ID living in group homes have a physical activity level (PAL) or ratio of 24 h energy expenditure to basal (often measured as resting) metabolic rate of around 1.2–1.4 [4,18]. This equates to either bed rest (PAL 1.2) or a sedentary (1.4) level [48]. Therefore, in this study, the energy intake of participants was compared to the Australian NRV for energy at a PAL of both 1.2 (bed rest) and 1.4 (sedentary). This was considered the most likely activity range for the participants and also at the lowest end of expenditure more likely to be consistent with lower resting energy expenditure. Unfortunately, it was beyond the scope of this study to directly measure or estimate energy expenditure of the participants.

### 2.5. Statistical Analysis

Data are reported as the mean ± standard deviation (SD), 95% confidence interval (CI) or proportion (%) of participants with intakes less than the EAR or RDI/AI (or more than the Upper Limits (ULs)). Participants were also categorised into two groups in relation to micronutrient intake: 1. those who achieved all or all but one of the EARs for selected micronutrients and 2. those not meeting the EAR for two or more of the selected micronutrients. Statistical analysis was performed using Statistical Package for the Social Sciences (SPSS) (version 22) and Graphpad Prism 6 by GraphPad Software. Comparison between men and women for micronutrient intake was conducted using independent t-tests. One-sample t-tests were used to examine the difference between serves of intake and recommended number of serves for adults from the AGHE [40]. A *p*-value of < 0.05 was set as the level of significance.

## 3. Results

### 3.1. Participant Recruitment, Demographic and Anthropometric Characteristics

Of the three not-for-profit organisations approached, two agreed to participate in the study. These organisations ran 59 group homes servicing a total of 283 people with ID and other mental health disorders in the metropolitan area covered by the study. Nine group homes from these two organisations that were within the research area (Sydney metropolitan city) agreed to participate, preliminary meetings revealed that only seven fitted the inclusion criteria. The two group homes that were excluded had residents that were more independent and did not require a carer’s assistance in planning and preparing their menu.

A convenience sample of 38 people (men (M): 16; women (W): 22) was recruited—of these, five people were later excluded due to them being more independent in providing their own meals or the participants were eating out frequently with family/friends during the collection period, resulting in only one day of measured food intake being collected. This resulted in a final sample of *n* = 33 (M: 14; W: 19) participants. Of these, food intake data were collected on three days for most participants (*n* = 28) and two days for five participants. The participant demographic characteristics are summarised in Table 1.

A slightly higher proportion of women to men (58% women) were recruited. Most participants (52%) were over 50 years, although two participants were in their early 20s. The mean BMI of all participants was 27.5 ± 6 kgm^−2^ (M: 25.6 ± 4 kgm^−2^; W: 28.9 ± 7 kgm^−2^). Forty-two percent were classified as having a moderate level of ID with 36% having severe ID. Eighty-two percent were independently mobile and only one participant was wheelchair dependent (Table 1).

Observation and weighing of participant intakes were primarily conducted by the research dietitian (NNH). The recording of 17% of dietary records (mainly supper) was assisted by the carers who were briefed prior to the data collection. A small number (*n* = 2; 6%) of participants were provided with a commercial supplemental formula (less than 500 mL) and some people required texture modified foods (*n* = 9; 27%) or assistance to eat at meals (*n* = 4; 12%).

### 3.2. Adequacy of Dietary Intake against Nutrient Reference Value

#### 3.2.1. Energy and Macronutrient Intake

The energy and macronutrient intake of participants is summarised in Table 2. Only some of the participants (33%; M: 17%; W: 47%) consumed an energy intake which met or exceeded the Australian NRV for energy requirements based on a PAL of 1.4. A higher proportion (64%; M: 29%; W: 89%) met or exceeded the energy requirement recommended for bed rest (PAL of 1.2). There was no statistical difference in energy intake between men and women (*p* = 0.46); additionally, the difference per kg of body weight was negligible (1 kJ/kg). The macronutrient intakes were generally within the Australian Acceptable Macronutrient Distribution Range (AMDR) except for fat. The mean total fat intake was 32.6 ± 9.6% and 35.8 ± 7.6% of energy for men and women respectively. This was at the upper end of the Australian AMDR of 20%–35% [43], especially for women. Sixty percent of participants (M: 57%; W: 63%) consumed above 35% of energy from fat. The mean proportion of energy from saturated fat was 13.3 ± 6.1% (M: 11.9 ± 5.3%; W: 14.9 ± 5.2%). A substantial proportion (M: 64%; W: 78%) of the participants consumed more than the Australian AMDR recommendation of 10% of total energy intake from saturated fat [43]. No participants consumed below the AMDR or less than 20% of energy from fat. Nevertheless, the fat intake was relatively within the recommendation in absolute terms.

The mean intake of sugars was 21.1 ± 10.8% and 16.8 ± 6.6% of energy in men and women respectively. The mean ± SD dietary fibre intake was 23.0 ± 4.8g and 20.9 ± 5.9g for men and women respectively. All men and 94% of women reported a fibre intake below the Suggested Dietary Target (SDT) of 38 g for men and 28 g for women. None of the participants reported consumption of alcohol on the days measured.

#### 3.2.2. Micronutrient Intake

The micronutrient intake of participants is summarised in Table 3. The proportion of individuals consuming below the EAR for specific nutrients ranged from 6% (riboflavin) to 86% (magnesium) for men, and 5% (iron) to 78% (calcium) for women. Only 18% of participants met all EARs. The RDIs for magnesium and potassium were not met by any of the men. However, all participants exceeded the AI and most (59%) exceeded the UL for sodium.

### 3.3. Adequacy of Dietary Intake against the Australian Guide to Healthy Eating

#### 3.3.1. Intake of Core Food against Australian Guide to Healthy Eating

**Figure 1:**
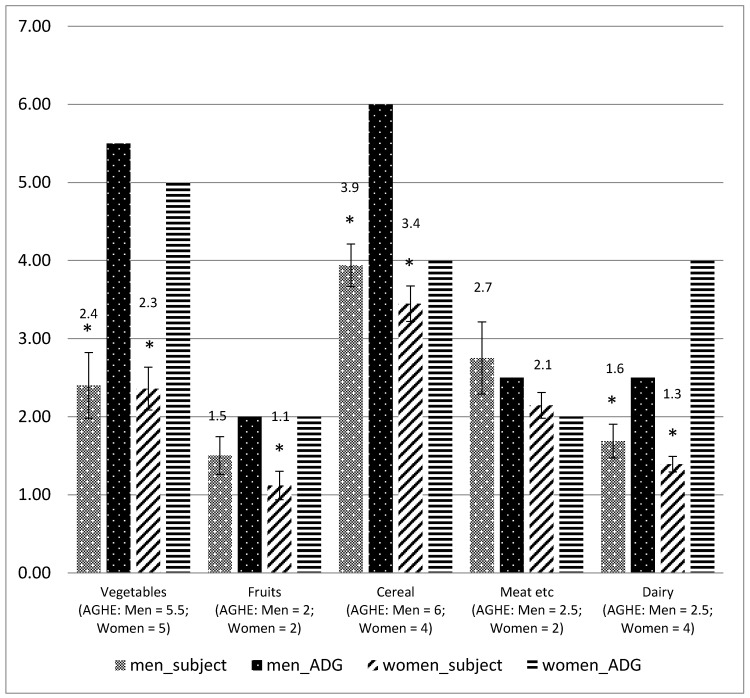
Serves of core food groups among men and women with ID, compared to the Australian Guide to Healthy Eating [50]. * *p* < 0.05 (one sample *t*-test).

The intake of foods was grouped according to food groups and the average number of serves compared to the AGHE (Figure 1). The recommended number of serves was not achieved except for the meat and meat alternative group (Figure 1).

Reported fluid consumption was <2 L/day, which was lower than the fluid NRVs (2.8 L for men and 2.1 L for women). This included plain drinking water, hot (tea/coffee) and cold beverages (cordials, fruit juice, and milk and milk alternatives, including milk used on cereal). These cereals generally include all types of bread, pasta, noodles, rice and other wholegrain and refined cereals.

#### 3.3.2. Intake of Discretionary Food against Australian Guide to Healthy Eating

**Figure 2:**
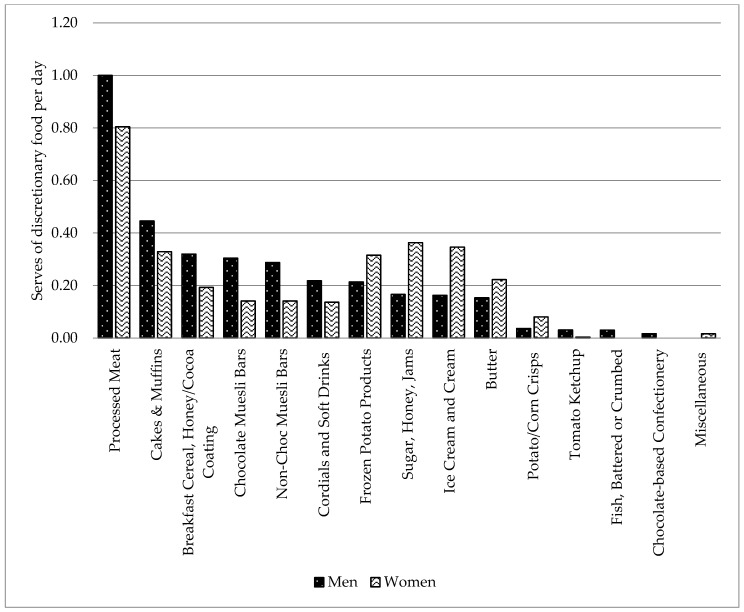
Serves of discretionary food items among men and women with ID.

Discretionary food consumption was 3.22 ± 0.21 serves per day, with men 3.38 and women 3.09 serves per day. Foods contributing most to the discretionary category were processed meat (predominantly ham), potato products (predominantly fries), sugary breakfast cereal (cocoa or honey coated), sweetened and savory biscuits, cakes, muesli and cereal style bars (chocolate coated or non-chocolate coated), sugar, honey, jam, and cordial (regular and low kilojoule varieties). Figure 2 shows serves per day consumed for several types of discretionary food by men and women from the study.

## 4. Discussion

To our knowledge, this study is one of the first to assess dietary intakes using a detailed dietary assessment method, in individuals with ID living in group homes in Australia. The results indicate that the diets of the participants were generally poor. More than 40% of the participants had micronutrient intakes well below the EAR for key nutrients including calcium, magnesium and iodine and for men, zinc. The average energy intake of the participants was (~7000–7400 kJ/d) similar to the energy requirements estimated for bed rest. A substantial proportion of the participants also consumed more than 10% of energy from saturated fat. Dietary fibre intake was below the Suggested Dietary Target for 92% of the sample. Analysis of servings from the different food groups indicated that the intake of vegetables was less than half of the Australian Guide to Healthy Eating (AGHE) and this was also the case for dairy foods in women. Intake of discretionary foods was relatively high, with processed meats being a common discretionary food. Overall, the dietary intake of the participants was not consistent with good health recommendations.

### 4.1. Energy Intake

The energy intake of the participants in this study was low and similar to bed rest (Table 2). When compared to other studies [4,31,51,52,53] which had measured the energy intake of people with ID, the mean energy intake in our study (7.2 MJ) is similar to that of Bertoli et al.’s [16] (7.3 MJ), which also assessed resting energy expenditure via indirect calorimetry in people with ID. There is a limited body of literature investigating the energy requirements for people with ID. This may reflect the diversity of diagnosis among people with ID, and the challenging environment to conduct this kind of research. It is widely reported that the prevalence of obesity among people with ID is higher than the general population [27,30,52].

In predicting the energy requirements of people with ID, there is evidence that people with ID have a lower resting metabolic rate. However, when adjusted for lean mass, resting metabolic rate has also been shown to be consistent with predicted values [18]. Research to determine the energy requirements of people with ID more accurately, would help guide menu planning which would aim to provide adequate nutrients within the relevant energy requirements for this population. It would also assist with promotion of enabling physical activity in residents. Group homes could be a suitable location to initiate physical activity programs. Individuals with ID may find it more challenging to exercise for a number of reasons: being less independent; decreased coordination; other physical limitations associated with their condition; and, potentially for some, the social stigma of exercising in environments such as gyms or recreation centres [43,52,53,54,55]. Even simple interventions such as daily walking with a carer will increase energy expenditure, which, in turn, would reduce the risk of weight gain [56].

### 4.2. Macronutrient Intake

The mean macronutrient composition in this present study was within the Acceptable Macronutrient Distribution Range (AMDR) and similar to the recent (2011–2012) Australian Health Survey (AHS), and other published findings on the dietary intake of adults with ID [18,31,57]. Our study found low fibre intake in people with ID and this is consistent across many studies in this population [4,18,31,41,57]. Lower fibre intake is a result of lower intake of wholegrain breads and cereals, fruits and vegetables (Figure 1). Low fibre intakes are also associated with increased risk of inadequate micronutrient intake, including thiamin, magnesium, phosphorus, vitamin C and folate (Table 3). Many people with ID experience constipation which can be mitigated, if not prevented, by an adequate intake of dietary fibre and fluids [58]. The capacity of diet to improve this aspect of care of people with ID has not been realised widely in the group home setting.

### 4.3. Micronutrient Intake

Micronutrient intakes of participants in this study were poor. Magnesium, calcium and iodine were below the EAR in a substantial proportion of participants (>43%) and for men, a substantial proportion (43%) had intakes below the EAR for zinc. Although intakes below the EAR do not necessarily indicate deficiency, the EAR is only adequate for 50% of the Australian population and so intakes below this level increase the likelihood of nutrient deficiency. Studies reporting on the micronutrient intake of people with ID are limited but others have reported marginal micronutrient intakes in this population [18,31]. This is particularly the case for calcium which was below the EAR in 43% and 78% of men and women in this study and this is consistent with the findings of other studies [18,31]. As osteoporosis is a common condition in people with ID, our finding of marginal calcium intake amongst our participant is clinically significant [59,60,61,62].

While the participants’ intake of micronutrients such as calcium was inadequate, their intake of sodium was the reverse. Sodium intake exceeded the recommended upper limit (UL) in more than half (59%) the participants. This is similar to the sodium intake reported in the general population, where two-thirds of the population (76% of males and 42% of women) consumed more sodium than recommended [63]. As residents of Australian group homes are not provided table salt, the sources of the high sodium intake were mostly associated with the high processed meat (predominantly ham) intake, cereals and breads. Some was also obtained from salt added in cooking. Excess salt is a risk factor for hypertension and cardiovascular disease for those who are in overweight and obese category [64,65]. Hence, strategies to reduce sodium intake, in particular from discretionary food choices, and limiting the addition of salt to food preparation is important [40].

Saturated fat was consumed in amounts greater than recommended by a substantial proportion (72%) of the participants (Table 2). Consistent intake of saturated fat above the recommended guidelines increases the risk for cardiovascular disease. Other studies in people with ID have also identified higher than recommended saturated fat intake [18,41,51,57]. In this study, saturated fat came mainly from discretionary foods (particularly processed meat).

### 4.4. Intake of Servings According to the Australian Guide to Healthy Eating

Except for the meat and meat alternative category, the daily intake of other food groups (vegetables, fruit, grains, and milk) were lower than Australian recommendations [40]. The low vegetable intake in particular was a concern as consumption was less than half of recommended guidelines [40] in both men and women (<2.5 serves per day instead of 5). When compared to result from the Australian Health Survey 2011–2012, vegetable intake from both men (2.40 serves per day) and women (2.36 serves per day) from this study was lower than intake of Australian adult aged 19 years and older (i.e., 3.0 serves per day; men 3.1, women 2.9 serves per day). Intake for fruits among men with ID (1.5 serves per day) was consistent with the intake of Australian adult aged 19 and older (1.5 serves per day). However, lower fruit intake for women with ID (1.12 serves per day) was observed when compared to the report from AHS 2011–2012 (1.40 serves per day).

This finding is consistent with a study in people with ID living in group homes in Sweden [4] and North America [41]. Individuals entering the group home environment do so with established dietary habits from childhood and so it may be difficult for carers to change these habits in the group home setting. Changing dietary behaviour may pose to be more difficult in people with ID as they have less ability to understand the importance of the suggested changes. It may be more difficult to introduce new foods and will be reliant on staff knowledge and motivation [32].

Another concern with the food group intake was an inadequate intake of dairy foods, especially in women. When compared to the population intake (AHS 2011–2012 report), dairy intakes for men with ID were higher (1.69 serves per day) whereas a similar intake (1.39 serves per day) was observed for women with ID (i.e., 1.4 serves per day; with men 1.5 serves per day, women 1.3 serves per day). However, when comparisons were made with the recommendations for dairy intakes, both men and women were low from the suggested servings of 2.5 serves per day for men and 4.0 serves per day for women. This was reflected in the substantial proportion (78%) who failed to meet the EAR for calcium. Given the higher prevalence of low bone density and osteoporosis in people with ID [60,61,62], this is concerning.

### 4.5. Strengths and Limitations

This study has a number of strengths, which includes measurement of dietary intake using a proxy-reporter method and photographed assisted observation. The direct observation and weighing of food enabled a detailed analysis of dietary intake to occur. A qualified dietitian also performed dietary analysis, and this enhanced its validity. Adjustment of the intakes to account for food fortification in Australia enabled micronutrient intake to be assessed more accurately. However, this study had a number of important limitations including the assessment of only three days of dietary intake in a small sample of people with ID living in not-for-profit organisation group homes. Although the people with ID and the days assessed were selected to represent the typical intake within a group home, analysis of the foods consumed over a longer period and in a wider number of participants in both government and non-government group homes may alter outcomes. This is particularly the case for micronutrients where a longer time period of collection is generally recommended. A longer recording period in a wider number of people with ID is needed to confirm our results. Validation of nutrient intakes using biomarkers is also recommended [66]. Not all nutrients were assessed including trans fats and the relative ratios of omega6:omega3 fatty acids, Vitamin B12 and Vitamin D. This was due to either limitations in the details provided from the nutrient database or priority to other nutrients which were considered more at risk in the diets of the population. Vitamin B12 is rarely low in diets containing animal flesh [67] and food provides small contribution to overall Vitamin D requirements in Australia; only a small portion of foods are fortified with Vitamin D (unlike some other countries, particularly Northern Hemisphere). Sunlight exposure makes an important contribution to the Vitamin D status of Australians [68].

Although a dietitian directly assessed intake, there were periods when the carers reported the intakes and it is possible that this resulted under- or misreporting. The low energy intake of the participants despite a high proportion being above the healthy weight range may raise concerns about the accuracy of the energy intakes. It is also possible that the participants consumed less on the days where the food intake was measured. Change in body weight was also not assessed to confirm weight stability. Unfortunately, it was beyond the scope of the study to assess energy expenditure or physical activity (e.g., doubly labelled water, resting metabolic rate, accelerometers, activity diaries).

It is possible that some participants used vitamin and mineral supplements, but that was not recorded. This is because the aim of our study was to investigate the nutritional composition of the food/diet of the residents which should for most of them provide an adequate intake of nutrients without the need of specific supplements. Commercial supplemental formulas provide energy and other macronutrients and so these form part of the ‘diet’ energy and were therefore treated as ‘food’ consumed in the analysis (e.g., if a resident had a small appetite and/or required supplementary feeding). While some residents for specific medical reasons may need additional ‘pill’ forms of vitamin/mineral supplements, the aim of residential care menus should be to provide good quality food that can meet the nutritional requirements of residents, even in the context where people may have a poor swallow and require texture modification, such as a soft diet. Adding in the supplements may have made the intakes appear adequate when in reality, the component provided from food was not.

### 4.6. Nutrition Recommendations

Although it would be important to address the overall diet of residents in group homes, it was evident that the types of snacks offered to the participants including cereal bars, biscuits, cake, and muffins could be replaced more often with fresh fruit, yoghurt, wholegrain cereal and milk or wholegrain bread or crisp bread and cheese. These options would be more filling, less expensive and would be more nutrient dense (particularly for the nutrients most commonly below the EAR; fibre, calcium, iodine and magnesium). Grated vegetables in meals and vegetable sticks could be offered with snacks to boost consumption. These simple strategies would also help to reduce the intake of less nutrient dense, discretionary foods. The use of water rather than cordial would also be recommended. Another simple suggestion to improve nutrient intakes would be to only purchase iodised salt for use in the home (although use should not be excessive to keep sodium intake within limits). A small proportion of the participants had an intake of Vitamin C below the EAR and this could be supported by only purchasing Vitamin C-fortified fruit juice, though frequent consumption of fruit juice is not recommended—rather, promote and provide readily accessible fruit in season.

## 5. Conclusions

This study found that the diets of the participants with ID living in group homes were not consistent with dietary guidelines. The intake of several key nutrients including, calcium, magnesium and iodine (and zinc for men) were below the EAR for a substantial proportion of the participants. Mean saturated fat intake was above and dietary fibre intake below the Suggested Dietary Targets set to reduce the risk of chronic disease. This study only measured the dietary intake of a small number of people with ID living in not-for-profit organisation group homes. While the results need to be replicated in a larger study across government and non-government homes, our findings raise concerns regarding the quality of the diets offered to people with ID in this setting and provide a platform for further investigation. As people with ID are at an increased risk of diet-related diseases such as obesity, type 2 diabetes, cardiovascular disease and osteoporosis, it is important for these dietary shortcomings to be addressed so that people with ID in group homes have the best chance to live a healthy life.

## Figures and Tables

**Table 1:** Demographic characteristics of participants.

Total Sample (*n* = 33)		
Classification	Men (*n* = 14) *n* (%)	Women (*n* = 19) *n* (%)
^a^**Age (years)**, mean ± SD (median)	48 ± 9 (50)	53 ± 17 (58)
19–30	1 (7)	2 (11)
31–50	7 (50)	6 (32)
51–70	6 (42)	10 (53)
>70	0	1 (5)
^b^**Body Mass Index** (kgm^−2^), mean ±SD (median)	26 ± 4 (27)	29 ± 7 (28)
18.50–24.99 (normal weight)	5 (36)	7 (37)
25.00–29.99 (overweight)	8 (57)	5 (26)
30.00–40.00 (obese)	1 (7)	7 (37)
^c^ **Disability Level**		
Mild	1 (7)	2 (10)
Moderate	6 (42)	8 (42)
Severe	6 (42)	6 (32)
* Profound	1 (7)	3 (16)
^d^ **Texture Modification**		
Normal	11 (79)	13 (68)
Soft	1 (7)	4 (21)
Minced and Moist	2 (14)	2 (10)
Smooth Pureed	0	0
**Mobility**		
Independent	11 (79)	16 (84)
Dependent/Assisted	2 (14)	3 (16)
Wheelchair dependent	1 (7)	0
**Liquid Meal Supplement Used**		
Yes	2 (14)	0
No	12 (86)	19 (100)

SD standard deviation; ^a^ based on classification by NHMRC: National Health and Medical Research Council; ^b^ Adapted from World Health Organisation (WHO) 1995, 2000 and 2004; ^c^ DSM-IV-TR: The Fourth Edition of the Diagnostic and Statistical Manual of Mental Disorders—Text Revision; ^d^ Australia Standards for Texture Modified Foods and Fluid [49] * All individuals with a profound intellectual disability were spoon fed.

**Table 2:** Energy and macronutrient intake among men and women with intellectual disability.

Macronutrients	Mean ± SD [95% CI]		Median (Inter-Quartile Range)	
	Men (*n* = 14)	Women (*n* = 19)	Men	Women
**Energy (kJ per day)**	7439 (6676–8202)	7034 (6461–7607)	7365 (5520–9134)	6846 (5444–9797)
kJ/kg	101.8 ± 30.2 (95.2–137.7)	111.2 ± 27.8 (92.2–136.1)	102.5 (82.4–197.9)	113.9 (55.0–165.1)
**% ≥PAL 1.2 ^a^**	29	89		
**% ≥PAL 1.4 ^a^**	17	47		
**Carbohydrate total (g/d)**	206.2 ± 55.2 (174.1–237.9)	187.2 ± 55.2 (162.6–211.8)	203 (130–332)	180 (108–302)
Recommendation	45%–65% of energy	45%–65% of energy		
% of energy	47.6 ±11.1	46.5 ± 9.7		
g/kg/d	3.2 ±1.3	3.2 ± 1.2		
**Total sugars (g/d)**	81.5 ± 32.1 (62.2–106.0)	77.8 ± 30.9 (59.0–103.1)	79 (38–146)	70 (28–139)
Recommendation	<10% of energy	<10% of energy		
% of energy	21.1 ± 10.8	16.8 ± 6.6		
**Protein (g/d)**	89.5 ± 15.5 (80.6–98.4)	75.6 ± 17.2 (63.5–97.1)	93 (61–118)	75 (47–105)
Recommendation	15%–25% of energy	15%–25% of energy		
% of energy	17.3 ± 2.4	17.2 ± 2.2		
g/kg/day	1.3 ± 0.3	1.1 ± 0.3		
**Fat total (g/d)**	63.9 ± 23.2 (50.6–77.3)	66.1 ± 11.6 (60.1–71.1)	67 (27–100)	66 (42–87)
Recommendation	20%–35% of energy	20%–35% of energy		
% of energy	32.6 ± 9.6	35.8 ±7.6		
**Saturated fat (g/d)**	24.2 ± 10.7 [14.1–27.2]	25.6 ± 8.0 [15.2–28.9]	26 (7–40)	30 (13–41)
Recommendation	<10% of energy	<10% of energy		
% of energy	11.9 ± 5.3	14.9 ± 5.2		
g/kg/day	[8.8–14.9]	[12.4–17.5]		
**Dietary fibre (g/d)**	23.0 ± 4.8 (19.3–24.6)	20.9 ± 5.9 (18.0–23.8)	24 (18–31)	21 (12–33)
Recommendation	30 g per day	25 g per day		

^a^ PAL: Physical Activity Level; 1.2—bed rest; 1.4—very sedentary (NHMRC, 2013).

**Table 3:** Micronutrient intake among men and women with intellectual disability.

Micronutrient	Mean ± SD [95% CI]		*p*-Value	^a^ EAR		^a^ RDI/^a^ AI		% < EAR		% < RDI/AI	
	Men (*n* = 14)	Women (*n* = 19)		M	W	M	W	M	W	M	W
**Thiamin (mg/d)**	1.7 ± 0.6 (1.4–2.1)	1.7 ± 1.1 (1.2–2.2)	0.929	1.0	0.9	1.2	1.1	7	21	13	26
**Riboflavin (mg/d)**	2.2 ± 0.6 (1.9–2.5)	2.2 ± 1.0 (1.8–2.7)	0.945	1.1	0.9	1.3	1.1	6	0	6	0
**Niacin Eq (mg/d)**	44.6 ± 7.7 (40.2–49.1)	39.9 ± 8.7 (35.7–44.0)	0.109	12	11	16	14	0	0	0	0
**Vitamin C (mg/d)**	111.7 ± 47.2 (84.5–138.9)	94.4 ± 58.4 (66.2–122.5)	0.369	30	30	45	45	0	7	0	13
**Vitamin E (mg/d)**	6.9 ± 2.0 (5.8–8.1)	6.0±2.5 (4.7–7.2)	0.219	-	-	^c^ 10	^c^ 7	-	-	93	89
**^b^ Folate (µg/day)**	531.1 ± 137.7 (451.5–610.5)	572.1 ± 302.4 (426.3–717.8)	0.640	320	320	400	400	0	16	13	26
**Magnesium (mg/d)**	295.9 ± 51.6 (266.1–325.6)	260.0 ± 76.5 (223.1–296.8)	0.139	350	265	420	320	86	63	100	78
**Calcium (mg/d)**	763.2 ± 321.8 (577.4–949.0)	709.6 ± 211.2 (607.9–811.4)	0.593	840	1100	1000	1300	43	78	86	94
**Phosphorus (mg/d)**	1384.5 ± 242.1 (1244.7–1524.2)	1272.3 ± 313.7 (1121.1–1423.5)	0.256	580	580	1000	1000	0	0	26	26
**Iron (mg/d)**	12.6 ± 4.0 (10.3–15.0)	10.1 ± 3.5 (8.4–11.7)	0.065	6	5	8	8	0	5	12	42
**Zinc (mg/d)**	13.7 ± 3.9 (11.5–16.0)	10.1 ± 2.5 (9.0–11.4)	0.007	12	6.5	14	8	43	0	66	21
**Iodine (µg/d)**	136.9 ± 48.6 (108.8–164.9)	128.6 ± 39.0 (109.8–147.4)	0.593	100	100	150	150	43	47	67	74
**Sodium (mg/d)**	2565.7 ± 981.1 (1999.2–132.1)	2404.5 ± 1241.6 (1806.1–3002.2)	0.691	-	-	^c^ 460–920	^c^ 460–920	-	-	0	0
**Potassium (mg/d)**	2774.4 ± 458 (2510.1–3038.7)	2417.3 ± 908.7 (1979.3–2855.3)	0.188	-	-	^c^ 3800	^c^ 2800	-	-	100	79

M: Men; W: Women; ^a^ EAR (Estimated Average Requirement), RDI (Recommended Dietary Intake) and AI (Average Intake) among men and women at 50–70 years; ^b^ Folate = total dietary folate equivalent; ^c^ Adequate Intake (AI). EAR and RDI for other age categories as follows. Men: Riboflavin: EAR > 70 (1.3 mg/day), and RDI > 70 (1.6 mg/day); Magnesium: EAR 19–30 yr (330 mg/day) and RDI 19–30 yr (400 mg/day); Calcium: EAR 19–70 yr (840 mg/day) and RDI > 70 yr (1300 mg/day).Women: Riboflavin: EAR > 70 yr (1.1mg/day) and RDI > 70 yr (1.3 mg/day); Magnesium: EAR 19–30 yr (255 mg/day) and RDI 19–30 yr (310 mg/day); Calcium: EAR 19–50 yr (840 mg/day) and RDI 19–50 yr (1000 mg/day); Iron: EAR 19–50 yr (8 mg/day) and RDI 19–50 yr (18 mg/day).
